# Predictors of return to work among mine workers following on-duty injury: A scoping review

**DOI:** 10.4102/sajp.v81i1.2165

**Published:** 2025-06-30

**Authors:** Wilinda Botha, Nontembiso Magida, Witness Mudzi, Bashir Bello

**Affiliations:** 1 Department of Physiotherapy, School of Health Care Sciences, Faculty of Health Sciences, University of Pretoria, Pretoria, South Africa; 2 Department of Physiotherapy, Faculty of Health Sciences, University of the Witwatersrand, Johannesburg, South Africa; 3 Department of Physiotherapy, Faculty of Allied Health Sciences, Bayero University Kano, Kano, Nigeria; 4 Lifestyle Disease Research Entity, North-West University, Mafikeng, South Africa

**Keywords:** return to work, occupational injury, rehabilitation, injured on duty, predictors, mine-workers

## Abstract

**Background:**

Mining industry workers face substantial challenges returning to work after occupational injuries. Despite the critical importance of successful return to work (RTW) outcomes, there is limited understanding of specific predictors within the mining sector that influence these outcomes.

**Objectives:**

To systematically identify and analyse factors influencing RTW among mine workers following on-duty injuries and synthesise evidence to inform rehabilitation strategies.

**Method:**

We searched PubMed, CINAHL, Google Scholar and PsycINFO databases for literature published between 1996 and December 2024. Studies were screened using predetermined inclusion and exclusion criteria. Data from eligible studies were extracted and analysed thematically to identify key predictors of RTW outcomes.

**Results:**

Three studies met the inclusion criteria. Negative RTW predictors (barriers) included physical complications (muscular atrophy, chronic pain), psychological factors (work avoidance, secondary gains) and workplace challenges (harsh conditions, job stress). Positive predictors (facilitators) encompassed supportive work environments, shorter rehabilitation periods, and financial stability. Demographic characteristics, particularly younger age and higher education levels, were associated with improved RTW outcomes.

**Conclusion:**

Return to work success among mine workers is influenced by multiple interconnected factors spanning physical, psychological, workplace and demographic domains.

**Clinical implications:**

These findings emphasise the need for comprehensive rehabilitation programmes integrating medical care, psychological support and workplace modifications. Future research should focus on developing targeted interventions that address these multiple dimensions of RTW in the mining sector.

## Introduction

Workplace injuries predominantly occur while involving heavy equipment or hazardous tasks. While every profession entails some implicit risk of injury, the degree of such risk varies significantly across different occupations, sectors, geographical regions and individual circumstances (Abdalla et al. 2017). Occupational risk factors contribute globally to a total of at least 1.9 million deaths each year and account for 90 million disability-adjusted life years (Tamene et al. 2022). Notably, 40% of claims under the *Compensation for Occupational Injuries and Diseases Act (COIDA)* are categorised as originating from musculoskeletal (MSK) injuries (Ferreira & Strydom 2016). These MSK injuries, such as strains, sprains and fractures, typically require recovery periods of up to 12 weeks or more (Cancelliere et al. 2016), culminating in negative impacts on overall wellbeing and incurring substantial societal costs for numerous patients (Hutting et al. 2017).

In instances when an individual sustains an injury on duty (IOD), the return to work (RTW) in either pre-injury or adjusted roles is critical in disability prevention (Johnston et al. 2012). It is imperative to reintegrate patients into safe and productive work environments as promptly as feasible. Research indicates that patients who continue to engage in work and gradually increase their workload towards normality tend to experience expedited recovery (Tamene et al. 2022). Musculoskeletal injuries are often episodic and recurrent, contributing to prolonged claims duration (Maas, Koehoorn & Mcleod 2021). Increased absenteeism correlates with diminished RTW outcomes (Tamene et al. 2022). Reduced RTW outcomes are associated with higher absenteeism (Tamene et al. 2022), which may exacerbate the effects of other influencing factors that have a significant impact on RTW (Bai et al. 2022; Kus et al. 2022), including injury severity, location, physical impairments, personal and sociodemographic characteristics, and social and psychological factors.

The International Classification of Functioning, Disability, and Health (ICF) provides a framework in which structures, activities, functions and participation can be integrated with contextual factors, including personal and environmental considerations, to foster a holistic approach (Kus et al. 2022). The ICF concept could thus be aligned with the scoping review eligibility PCC framework, where the Population (P) refers to individuals with disabilities or health conditions (injured mine workers), Concept (C) means integration of structures, activities, functions and participation with contextual factors (predictors of RTW), and Context (C) refers to holistic approach to disability and health (those who have experienced an on-duty or workplace injury).

The impacts of duty-related injuries extend to bodily functions (pain and range of motion) and structures (joints or muscles), activities (self-care) and participation (daily functioning) (Kus et al. 2022). Factors such as restricted mobility, prior injuries and considerable pain complaints, including referrals to the limbs, adversely affect RTW outcomes (Storheim et al. 2005). Individuals’ likelihood of returning to work improves when they can independently perform activities of daily living (Ntsiea, Van Aswegen & Olorunju 2013). In addition, general health and current health status serve as predictors for the timing of return to work (Kus et al. 2022). Other studies have indicated that higher baseline income and job involvement correlate with shorter RTW durations and pre-injury working hours (Murgatroyd et al. 2016). Some injured workers express reluctance to work while in pain, with union representatives advocating for the worker’s right to take leave post-injury (Tamene et al. 2022). Conversely, for certain employees, active involvement in work can provide therapeutic benefits. Various work-related predictors may influence an employee’s considerations regarding RTW.

Predictors related to environmental factors encompass professional sector dynamics, unresolved legal disputes and financial concerns (Kus et al. 2022). Delays in claims processing coupled with inadequate claim management practices contribute to adverse mental health and stress levels in injured workers (Collie et al. 2019). In the construction sector, roles that entail constant heavy lifting, workers’ compensation and insurance status, and limited occupational skills are negative predictors of RTW (Murgatroyd et al. 2016). Industries such as trade and manufacturing prioritise a gradual RTW process, in which access to resources and modified working hours are adjusted to support workers during their recovery (Maas et al. 2021). While employers may modify job-related factors, injuries can also influence personal factors. Personal predictors include personality traits, pre-injury life satisfaction, illness perception, low self-efficacy (Murgatroyd et al. 2016), attitudes towards life and the demand for pension claims (Kus et al. 2022). Factors such as marital status, family responsibilities (e.g., being the primary breadwinner) and young age contribute in varying ways to RTW (Weerdesteijn et al. 2020). Negative predictors include divorce, living alone, being female (Wong et al. 2021), older age, lower physical activity levels, having multiple children, fear-avoidance behaviours, low energy and low expectations for RTW (Hedlund et al. 2021). Return-to-work restoration and maintenance programmes have demonstrated positive effects, but severe depressive disorders can hinder work productivity and are linked to work limitations (Hoaki & Terao 2021). It is believed that employees’ motivation and attitude significantly influence their resistance to modified work; however, when workers perceive that their employers care about their wellbeing, they are more willing to cooperate (Rasool et al. 2021). Jobs with high psychological demands are associated with delayed RTW and may induce a fear of recurrent or exacerbated symptoms (Haveraaen, Skarpaas & Aas 2017).

Despite the significant impact of workplace injuries on individual health, productivity and societal costs (Hutting et al. 2017; Tamene et al. 2022), there remains a lack of consistent evidence regarding the factors predicting RTW following on-duty injuries among mine workers. Although research has identified various RTW predictors in other industries – spanning physical, personal and environmental factors (Bai et al. 2022; Kus et al. 2022) – the unique challenges and risks in the mining sector warrant a focused investigation. The lack of a comprehensive understanding of RTW predictors specific to mine workers limits the development of effective strategies to facilitate timely and successful RTW in this high-risk occupation (Maas et al. 2021). This scoping review seeks to address this gap by systematically reviewing and synthesising the available evidence on the predictors of RTW among mine workers following on-duty injury to inform improved practices for injury management and occupational rehabilitation within the mining industry.

## Research methods and design

This scoping review was conducted using the methodological framework established by Arksey and O’Malley (Levac, Colquhoun & O’Brien 2010), and the updated guidance from the Joanna Briggs Institute (JBI). This approach is particularly suited for exploring the breadth of literature surrounding the predictors of RTW among mine workers following on-duty injury (Khalil et al. 2024). The methodology consisted of the following key stages: identifying the research question, identifying relevant literature, selecting literature, charting the data and collating, summarising and reporting results.

### Identifying the research question

The primary research question guiding this scoping review is: ‘What are the predictors of RTW among mine workers following on-duty injury’? This question delineated the scope of the review and ensured that the literature search was focused and relevant.

### Identifying relevant literature

A comprehensive literature search was conducted across multiple databases, including PubMed, CINAHL, PsycINFO, Cochrane Library, Medline, Academic Search Complete, Free Medical Journals, LiLACS, ProQuest, SCOPUS, Cochrane Register of Systematic Reviews, PEDro and Google Scholar. The search strategy employed a combination of keywords and phrases such as (‘Return to work’ OR ‘Work reintegration’) AND (‘Mine workers’ OR ‘Miners’) AND (‘Occupational injury’ OR ‘Workplace injury’) AND (‘Predictors’ OR ‘Determinants’). The search was limited to peer-reviewed articles published in English from 1996 to December 2024 to ensure the inclusion of recent advancements in the field, and based on the amendment of COIDA in 1996.

### Selecting literature

The eligibility criteria were structured based on the PCC framework – Population (mine workers), Concept (predictors of RTW) and Context (those who have experienced an on-duty or workplace injury) – commonly used in scoping reviews. Inclusion criteria were established to guide the selection of relevant studies. Articles were included if they focused on mine workers, workers in the mining industry and adult participants (18–65 years) who have experienced an on-duty injury or workplace injury. Studies investigating predictors, determinants, barriers or facilitators of returning to work and the physical, psychosocial, workplace and economic predictors were included. Articles were excluded if they were reviews, editorials, commentaries or opinion pieces without empirical evidence or studies addressing general injury recovery without specific outcomes related to return to work. Articles focused on interventions unrelated to return to work outcomes were also excluded.

**FIGURE 1:**
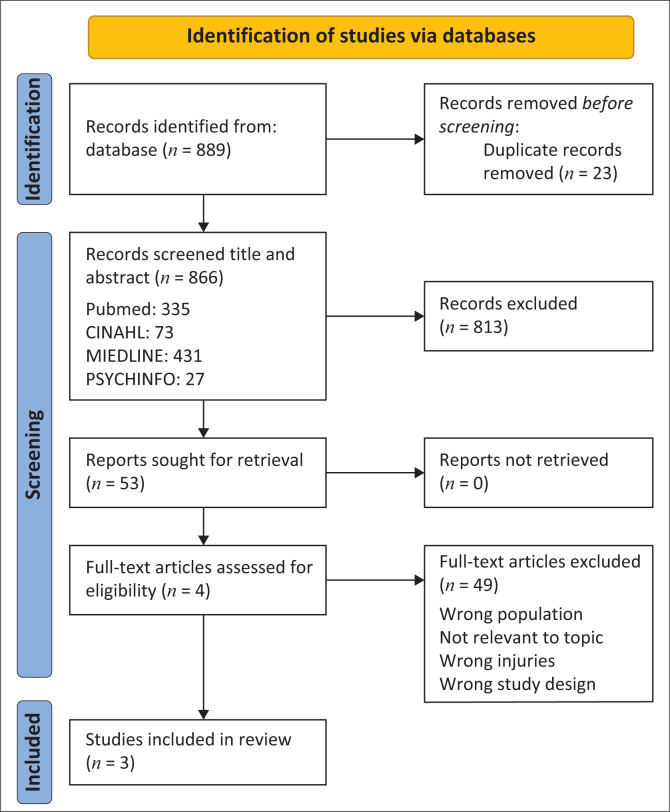
Preferred Reporting Items for Systematic reviews and Meta-Analyses (PRISMA) flow chart diagram.

The selection process was conducted in two phases: an initial screening of titles and abstracts and a full-text review of potentially relevant articles, as described by Khalil et al. (2024). Figure 1 illustrates the flowchart of the screening process.

### Charting the data

A data extraction form was created to capture key information from each included study systematically. The data included details of the author(s), year of publication, study design, sample size, injury type, predictors and/or determinants, time to RTW, duration of absenteeism, and others.

### Collating, summarising and reporting results

The final stage involved collating and summarising the findings from the charted data. The results were organised thematically based on the key areas of predictors of RTW, including demographic factors for RTW, health-related factors for RTW, psychological-related factors for RTW and work-related predictors for RTW.

### Ethical considerations

This article followed all ethical standards for a research without direct contact with human or animal subjects.

## Results

The scoping review aimed to identify predictors of RTW in mine workers post-injury on duty. Four electronic databases (PubMed, CINAHL, MEDLINE and PsycINFO) were systematically searched from 1996 to December 2024. A total of 889 records were obtained, and after duplicates were removed, 866 remaining records were screened based on title and abstract of which 813 were excluded. The 53 full-text articles were assessed for eligibility, and 49 articles were excluded for the incorrect population, study design, injuries and setting. Only 3 articles met the full eligibility criteria and were included in the review (Table 1). Our comprehensive scoping review uncovered a multifaceted landscape of factors influencing RTW among mine workers following workplace injuries. The identified predictors were systematically organised across negative and positive dimensions, encompassing demographic, health, psychological and occupational domains.

**TABLE 1:** The predictors of return to work in mine-workers post-injury on duty.

Authors and year	Negative predictors for RTW	Positive predictors for RTW	Demographic factors related to RTW	Health-related factors for RTW	Psychologically-related factors for RTW	Work-related predictors for RTW
Tiftikci et al. (2015)	Haematoma-effusion inside the knee Muscular atrophy Long duration of the rehabilitation therapy process The unwillingness of patients who undergo operations for work accidents to RTW	Secondary gain (such as other workplace benefits i.e., bonuses or promotion, etc.) The shorter duration of the rehabilitation therapy process Psychological support and physical rehabilitation therapy are similar to those of athletes Higher Lysholm, Cincinnati and Tegner activity scores postoperatively Criteria for returning to work based on knee function test, single-legged hop test score, Lachman test, anterior drawer test and pivot-shift test in miners	The average age of patients is 27.8 years, with all patients being male	Haematoma-effusion inside the knee Muscular atrophy Long duration of the rehabilitation therapy process Pain inside the knee	Post-traumatic stress disorder	Harsh working conditions
Bhattacherjee and Kunar (2016)	Presence of disease Job stress	Lower number of dependents Longer sleep duration Absence of job stress Absence of disease No alcohol addiction Higher monthly income	Age and education level Height and weight Total number of dependents Regular consumption of alcohol Tobacco use diseases Family size	Presence of disease Sleeping habit Injury type	Work stress and job satisfaction	Job stress Job satisfaction Monthly income Workplace variables such as supportive co-workers Seniority
Corbière et al. (2016)	Individuals’ overcommitment to their work, including high expectations of themselves, perfectionism and the need to control the situation Factors related to the attitudes and behaviours of supervisors and contact persons include immediate supervisors perceived as not credible, conflicting relationships with supervisors, supervisors’ unavailability and patronising supervisors Conflict and tensions within the team, conflicting relationships with colleagues and conflicting relationships with employees/subordinates are also negative predicting factors	Being a very in-demand/overly sustained work pace Must make/announce decisions which have a (negative) impact on others Significant changes in task(s) Lack of time, overly tight deadlines Absence of (or few) concrete results The organisation’s high standards Solicitation of clients Always being in the spotlight, no time to be alone	The study included 15 women and seven men, with two-thirds having a university degree Almost half of the participants were between 31 years and 45 years, and married About two-thirds of the participants worked 10 years or less within the organisation from which they were absent because of depression Twelve participants worked in the public sector, seven in the private sector, and three in non-profit organisations	Sleep problems, exhaustion/fatigue Weight loss Tremors Headache Loss of appetite Pain Nausea	Attitudes and behaviours of supervisors and contact persons Over-commitment of individuals in their work Relational factors with key stakeholders of the organisation Reactions of denial and confusion with physical symptoms can impact the return to work process	Work-related psychosocial risk factors Individual’s experience in employment The period preceding the sick leave of individuals who experienced depression Having an overly sustained workplace Frequent changes of immediate supervisors Lack of support Overly tight deadlines

Note: Please see full reference list of this article, Botha, W., Magida, N., Mudzi, W. & Bello, B., 2025, ‘Predictors of return to work among mine workers following on-duty injury: A scoping review’, *South African Journal of Physiotherapy* 81(1), a2165. https://doi.org/10.4102/sajp.v81i1.2165, for more information.

### Negative return to work predictors

Significant barriers to successful RTW emerged across multiple dimensions. From a health perspective, workers encountered substantial challenges, including knee effusions, muscle degradation, extended rehabilitation periods and persistent pain. Medical complications extended beyond physical symptoms, incorporating sleep disruptions, chronic fatigue, traumatic stress responses and various underlying health conditions, as documented by researchers Tiftikci et al. (2015), Bhattacherjee and Kunar (2016), and Corbière et al. (2016).

Psychological dimensions revealed complex impediments to recovery. Workers demonstrated pronounced reluctance to resume employment, exhibited secondary gain behaviours and displayed intense work-related preoccupations. Manifestations of denial, perfectionist tendencies and unrealistic self-expectations further complicated rehabilitation trajectories (Bhattacherjee & Kunar 2016; Corbière et al. 2016; Tiftikci et al. 2015).

Workplace dynamics introduced additional complexity, with harsh environmental conditions, elevated job stress, dysfunctional team interactions, supervisor conflicts, and restrictive operational timelines substantially undermining RTW potential.

### Positive return to work facilitators

Several key enablers counterbalanced negative factors and supported successful workplace reintegration. Health-related interventions with short rehabilitation protocols and comprehensive physical-psychological support programmes significantly enhanced RTW prospects. Workplace and psychological support mechanisms proved instrumental. Environments featuring empathetic supervisors, adaptable job structures, minimal occupational stress and elevated job satisfaction consistently correlated with improved RTW outcomes. Socioeconomic stability emerged as another critical predictor, with higher income levels and the absence of substance dependency as meaningful recuperation indicators.

### Demographic influences

Demographic characteristics demonstrated nuanced impacts on RTW trajectories. Younger workers, averaging 27.8 years, and those possessing advanced educational backgrounds exhibited higher reintegration rates. Family composition, dependent count and marital status introduced additional variability across the studied populations.

### Psychological dimensions

Psychological resilience emerged as a pivotal RTW determinant. Targeted interventions addressing psychological barriers, particularly overcommitment patterns and denial responses, proved strategically effective in mitigating rehabilitation obstacles.

### Occupational factors

Workplace ecosystem dynamics profoundly influenced RTW’s success. Adaptive job roles, supportive colleague networks and employment seniority positively correlated with reintegration. Conversely, frequent organisational restructuring, unsupportive management and systemic pressures consistently undermined recovery efforts. The investigation reveals RTW among mine workers as an intricate phenomenon shaped by interconnected health, organisational, psychological and personal factors. Effective strategies must, therefore, adopt holistic, personalised approaches prioritising workplace adaptability and comprehensive psychological support to optimise rehabilitation outcomes.

## Discussion

This scoping review comprehensively examined the multifaceted predictors influencing RTW among mine workers following on-duty injuries (Bhattacherjee & Kunar 2016; Corbière et al. 2016; Tiftikci et al. 2015). Our analysis reveals the complex interplay of health, psychological, workplace and demographic factors as predictors of return to work.

The review highlights the critical role of physical health in RTW outcomes. Knee-related injuries, muscular atrophy and prolonged rehabilitation processes emerged as substantial barriers to successful workplace reintegration. These findings align with existing literature emphasising the profound impact of physical limitations on occupational recovery (Kool et al. 2007). The variability in rehabilitation outcomes underscores the need for personalised medical interventions beyond standard treatment protocols. Secondary health complications, including sleep disturbances, fatigue and chronic pain, further complicate the RTW process (Storheim et al. 2005). Corbière et al. (2016) explained that complex processes and factors could affect the predictors of RTW of workers. These interconnected health challenges suggest that a holistic approach to worker rehabilitation is essential, addressing primary injury-related concerns and associated physiological and psychological sequelae (Pransky, Loisel & Anema 2011).

Psychological factors emerged as pivotal determinants of RTW success. The review revealed that workers’ mental resilience, coping mechanisms and psychological support are crucial in recovery (Murgatroyd et al. 2016). The factors influencing RTW after an injury or illness can be categorised as positive (facilitators) or negative (barriers) within the biopsychosocial model. Implications of the findings showed that a focus on multidisciplinary care, integrating physiotherapy, mental health support and vocational rehabilitation would help mine workers RTW faster and easier following injury on duty. However, Bhattacherjee and Kunar (2016) maintained that characteristics such as overcommitment, perfectionism and denial responses significantly impeded RTW processes. This finding emphasises the critical need for integrated psychological support within rehabilitation programmes (Loisel et al. 2005). The prevalence of post-traumatic stress disorder (PTSD) and work-related psychological strain highlights the importance of mental health interventions (Corbière et al. 2016). Young et al. (2005) affirmed that existing rehabilitation models may benefit from incorporating more comprehensive psychological support strategies that address clinical and occupational recovery aspects.

Workplace dynamics demonstrated a profound influence on RTW outcomes. Supportive work environments, characterised by flexible job roles, understanding supervisors and colleague support, significantly enhanced workers’ ability to RTW, as demonstrated by the findings of this study. Conversely, harsh working conditions, job stress and organisational pressures may appear as substantial barriers to successful reintegration (Krause et al. 2001). These findings underscore the need for workplace interventions beyond individual rehabilitation efforts (Bai et al. 2022). Franche et al. (2005) stated that organisations must develop adaptive, supportive frameworks that facilitate worker recovery and reintegration, including modified job roles, flexible work arrangements and comprehensive support systems.

Demographic factors introduced nuanced complexity to the RTW processes (Kus et al. 2022). Younger workers and those with higher education levels demonstrated more successful reintegration outcomes, highlighting the importance of individual characteristics in shaping recovery trajectories (Bhattacherjee & Kunar 2016; Tiftikci et al. 2015). These insights underscore the dynamic interaction between age, educational background and socioeconomic factors with injury recovery and workplace reintegration (Kus et al. 2022; Stergiou-Kita et al. 2014). Consequently, workplace interventions promoting a healthy return to work should adopt a holistic approach, addressing the multidimensional factors influencing RTW outcomes. This includes tailoring support to the specific needs of diverse demographic groups to foster equitable and effective reintegration strategies (Tamene et al. 2022).

### Limitations and future research

While this review provides valuable insights, several limitations must be acknowledged. The review found a paucity of literature on predictors of RTW among mine workers, with only three studies included. Our study demonstrated variability in methodological approaches, sample characteristics and contextual factors. The predominantly male sample and focus on specific injury types may limit the generalisability of findings. Hence, future research should address these limitations by:

1. Conducting intervention studies, with representatives across different mining contexts.
2. Developing standardised assessment frameworks for RTW predictors.
3. Exploring long-term outcomes of comprehensive rehabilitation interventions.
4. Investigating the efficacy of integrated health, psychological and workplace support models on RTW for mine workers.

### Practical implications

The findings have significant practical implications for occupational health professionals, employers and policymakers. A multifaceted approach to worker rehabilitation is crucial, encompassing personalised medical and psychological support, workplace accommodation and flexibility, comprehensive return to work programmes and ongoing monitoring and support systems.

## Conclusion

This scoping review sheds light on the intricate process of RTW among mine workers, revealing a complex interplay of factors. The study’s findings identify four key predictors of successful RTW: positive facilitators, psychological factors, health-related factors and workplace-related factors. By acknowledging and addressing the multifaceted nature of workplace injury recovery, stakeholders can develop comprehensive, supportive strategies to facilitate workers’ reintegration and promote long-term occupational well-being.
